# Long-term trends in the clinical management and outcomes of patients with gastroesophageal cancer in Norway

**DOI:** 10.2340/1651-226X.2025.43167

**Published:** 2025-04-15

**Authors:** Alexander Borthen Kolstad, Gabrielle Emanuel, Geir Olav Hjortland, Yngvar Nilssen, Maria Ulvestad, Ali Areffard, Eirik Kjus Aahlin

**Affiliations:** a Department of Gastrointestinal Surgery, University Hospital of North Norway, Tromsø, Norway; b Institute of Clinical Medicine, Faculty of Health Sciences, University of Tromsø, Tromsø, Norway; c Bristol Myers Squibb, Uxbridge, United Kingdom; d Department of Oncology. Oslo University Hospital, Oslo, Norway; e Cancer Registry of Norway, Norwegian Institute of Public Health, Oslo, Norway; f Bristol Myers Squibb, Lysaker, Norway

**Keywords:** stomach neoplasms, oesophageal neoplasms, neoadjuvant therapy, retrospective studies, epidemiology, survival analysis

## Abstract

**Background and purpose:**

Gastroesophageal cancers are highly prevalent internationally, with many patients diagnosed with metastatic disease, leading to challenging treatment and poor survival. This study uses real-world evidence from a population-level database to describe demographics, clinical characteristics, initial treatment patterns, and survival for patients with gastroesophageal cancer in Norway.

**Material and methods:**

Individual patient data was sourced from the Cancer Registry of Norway for patients diagnosed with oesophageal squamous cell carcinoma (ESCC), oesophageal adenocarcinoma (EAC), gastroesophageal junction cancer (GEJC), and gastric cancer from 2001 to 2021, with follow-up from diagnosis to death or last follow-up. Treatment patterns were captured from 2010 to 2022, defined as curative or palliative based on surgery, chemotherapy, and radiotherapy.

**Results and interpretation:**

The cohort included 14,334 Norwegian patients with gastroesophageal cancer; predominantly male, mean age 69–73 years, with a median follow-up of 9–11 months across cancer subtypes. Approximately 40% of patients received curative treatment, and multi-modality treatments increased for EAC, GEJC, and ESCC. Median survival ranged from 6 to 11 months for patients treated palliatively, and 17–95 months for those treated with curative intent. Interestingly, median survival was higher for patients with EAC and GEJC treated with neoadjuvant chemotherapy (86.1 and 75.1 months) versus neoadjuvant chemoradiotherapy (49.1 and 42.1 months), which was confirmed by a multivariate Cox regression model adjusted for age, sex, and disease stage.

This study demonstrates that multimodal treatment strategies, consisting of chemotherapy and surgery, may be associated with improved survival outcomes for gastroesophageal cancers. Future studies are required to identify optimum treatment strategies for gastroesophageal cancer subtypes.

## Introduction

In Norway, gastroesophageal cancer had a reported incidence of 870 new cases and prevalence of 3,376 patients in 2023 [1, 2]. The relative survival has steadily improved in oesophageal cancer, with a more gradual improvement in gastric cancer (GC) (1984–2023), and recent 5-year relative survival (2019–2023) lies at 24–30% for oesophageal cancer and 29–39% for GC [1, 2]. Incidence by gastroesophageal cancer subtype varies, with oesophageal adenocarcinoma (EAC) and oesophageal squamous cell carcinoma (ESCC) accounting for 75–80% and 20%, respectively, of oesophageal neoplasms in Norway (2016) [3].

Early-stage symptoms of gastroesophageal cancer are often non-specific, leading to most patients being diagnosed with metastatic disease, where treatment is challenging and often palliative [4–8]. Advances in our understanding of gastroeso-phageal cancer have transformed the treatment landscape for locoregional disease over the past two decades, including pivotal clinical trials for neoadjuvant chemoradiotherapy and perioperative chemotherapy [9–12]. which have subsequently become the standard of care [13, 14]. The CROSS trial demonstrated improved survival with neoadjuvant chemoradiotherapy and surgery versus primary surgery in patients with EAC, ESCC, and gastroesophageal junction cancer (GEJC) [11, 12]. The MAGIC trial, later followed by the FLOT4 trial, demonstrated improved survival with perioperative chemotherapy and surgery compared with primary surgery in patients with EAC, GEJC, and GC [9, 10, 15].

While treatment options for gastroesophageal cancer have expanded, there are limited published reports on how current treatments influence the long-term outcomes in the different gastroesophageal cancer subtypes. This study aims to utilise real world evidence (RWE) from a population-level database to describe patient demographics, clinical characteristics, initial treatment patterns, and survival of patients with gastroesophageal cancer in Norway.

## Material and methods

### Study population

#### Total cohort

**Figure 1:**
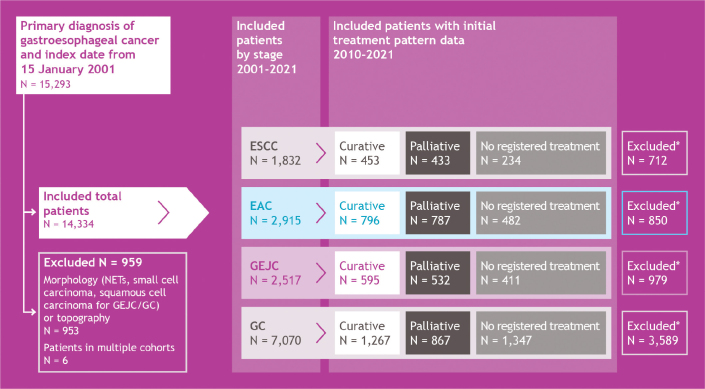
Cohort attrition. *Patients were excluded due to insufficient treatment pattern data available prior to 2010. EAC: oesophageal adenocarcinoma; ESCC: oesophageal squamous cell carcinoma; GC: gastric cancer; GEJC: gastroesophageal junction cancer; NET: neuroendocrine tumour.

Individual patient data was sourced from the Cancer Registry of Norway (including the Clinical Registry of Oesophagus and Gastric Cancer) and the Norwegian Patient Registry (NPR), and comprised pathology, age, sex, disease stage, Eastern Cooperative Oncology Group (ECOG) score, diagnostic/examination period, registration of treatment, and follow-up period [16–19]. Patients with a primary diagnosis of ESCC, EAC, GEJC, or GC from 2001 to 2021 were included in the cohort (Figure 1). Surgery was defined as surgical or endoscopic resection and did not include biopsy or surgical exploration without resection. The following codes were not included when defining surgery: 0, no surgery; 1, biopsy; 2, surgical exploration with or without biopsy; 95, biopsy from metastasis, local recurrence, or non-classified tumour; 96, cytology; and 99, no information about surgical procedure. Further details on topography and morphology codes used to define the populations are described in Supplementary Table S1.

Ethical approval was received from the Regional Ethics Committee (Reference: 339296).

Patients were included from the date of diagnosis, corresponding to the first appearance of a histologically verified cancer notification in the registry, and were followed until death, last follow-up, or administrative censoring for survival analysis (31 December 2021). A total of 15,293 patients initially fulfilled the inclusion criteria. However, some patients had multiple records with the same diagnosis date but differing International Classification of Diseases. 10th edition (ICD-10) or morphology codes. To prevent double counting, certain rules were applied where patients had multiple ICD-10 or morphology codes in their records with the same diagnosis date that would have categorised them in more than one cohort. Firstly, patients categorised as having GEJC and GC were classified as GC, as these patients most likely have Siewert type 3 GEJC cancer, which would be treated as GC. Secondly, patients categorised as having EAC and GEJC were classified as EAC to align with registry grouping. Thirdly, patients categorised as having EAC and GC were excluded to align with registry grouping, as it was not possible to identify the primary tumour, and these locations are too far apart anatomically to be treated similarly (*n* = 6 records). Finally, patients categorised as having GEJC and GC with morphology squamous cell cancer, small cell carcinoma, or neuroendocrine tumours were excluded (*n* = 953 records [366 due to neuroendocrine tumour codes]). The final total cohort comprised 14,334 patients from 2001 to 2021.

#### Initial treatment pattern cohort

Individual patient data on those who had received intravenous chemotherapy was obtained from the NPR, based on Norwegian Clinical Procedure Codes (NKPK) [18, 19]. Data on patients who received radiotherapy was obtained from the Cancer Registry of Norway (CRN) [16, 17]. Individual patient data on treatment patterns became available from 2008; as such, a period of 2010–2021 was chosen to capture sufficient data on initial treatment patterns, and patients prior to this date were excluded (Figure 1). The final initial treatment pattern cohort comprised 8,204 patients from 2010 to 2021. Initial treatment within 12 months of diagnosis was defined as curative (i.e. definitive chemoradiotherapy, neoadjuvant chemotherapy, neoadjuvant chemoradiotherapy, and primary surgery) or palliative (i.e. radiotherapy, chemotherapy, or chemoradiotherapy). Patients who received no surgery, chemotherapy, or radiotherapy were defined as no registered treatment.

For curative initial treatments, patients who received chemotherapy between the diagnosis and the surgery date within a year were defined as receiving neoadjuvant chemotherapy. Any treatment after surgery was allowed. The cohort was constrained to neoadjuvant chemotherapy, due to difficulties in the identification of patients who received chemotherapy within 3 months postoperatively to capture perioperative chemotherapy; also some patients may not have started adjuvant chemotherapy. Patients who received any dose of radiotherapy and chemotherapy between the diagnosis and surgery were defined as receiving neoadjuvant chemoradio-therapy. Patients who received ≥ 40 Gy of radiotherapy and chemotherapy within 1 year after diagnosis were defined as receiving definitive chemoradiotherapy.

For palliative initial treatments, patients who received < 40 Gy and chemotherapy within 1 year were defined as receiving palliative chemoradiotherapy. Patients who received no registered chemotherapy or radiotherapy between diagnosis and surgery within 1 year of diagnosis were defined as receiving primary surgery. Patients who received any amount of radiotherapy but no chemotherapy within 1 year after diagnosis were defined as receiving palliative radiotherapy. Further details on treatment definitions can be found in Supplementary Table S2.

Patients whose first treatment post diagnosis did not fall into the above categories were individually assessed and reassigned to the treatment group that most accurately reflected their clinical experience. Patients who received < 40 Gy of radiotherapy were re-assigned to neoadjuvant chemoradiotherapy (*n* = 8). Patients who received ≥ 40 Gy of radiotherapy before surgery, but did not have chemotherapy treatment recorded due to potential non-registration at the hospital, were re-assigned to neoadjuvant chemoradiotherapy (*n* = 13). Patients who did not receive chemotherapy and radiotherapy on the same date were re-assigned to definitive chemoradiotherapy (*n* = 146). Patients were re-assigned to the chemotherapy group who received radiotherapy after surgery within a year after diagnosis (*n* = 41); 33 patients received > 40 Gy, while 8 received < 40 Gy. This could be due to local recurrence, adjuvant chemoradiotherapy in connection with the CRITICS clinical trial [20], R1/R2 resection with subsequent radiation. Patients were excluded if they had received either chemotherapy, surgery and radiotherapy, or surgery and radiotherapy, or surgery and chemotherapy, on the same date (*n* = 11).

### Study variables and outcomes

A list of study variables and outcomes is provided in the Supplementary materials (Supplementary Table S3). The cancer stage at diagnosis is based on the staging according to the U.S. National Cancer Institute’s Surveillance, Epidemiology and End Results (SEER) program. Metastasis codes were grouped as localised (0 and 8 [node negative and T1–T3]), regional spread (A, D, 1, 5, and 6 [node positive and T1–T3, or T4 and/or node positive]), distant metastases (B, 2, 3, and 4 [metastatic disease, M1]), or unknown (C, 7, 9).

### Statistical analysis

Patient demographics and clinical characteristics were presented using descriptive statistics, with categorical variables presented as patient number and percentage, and continuous variables presented as median, mean, and range. Subgroup analyses included disease stage, age, and initial treatment. Kaplan–Meier analysis was conducted to estimate overall survival (OS), and Cox regression analysis was performed to adjust results against multivariates including age, sex, and disease stage.

## Results

### Patient demographics and clinical characteristics

#### Total cohort

**Figure 2:**
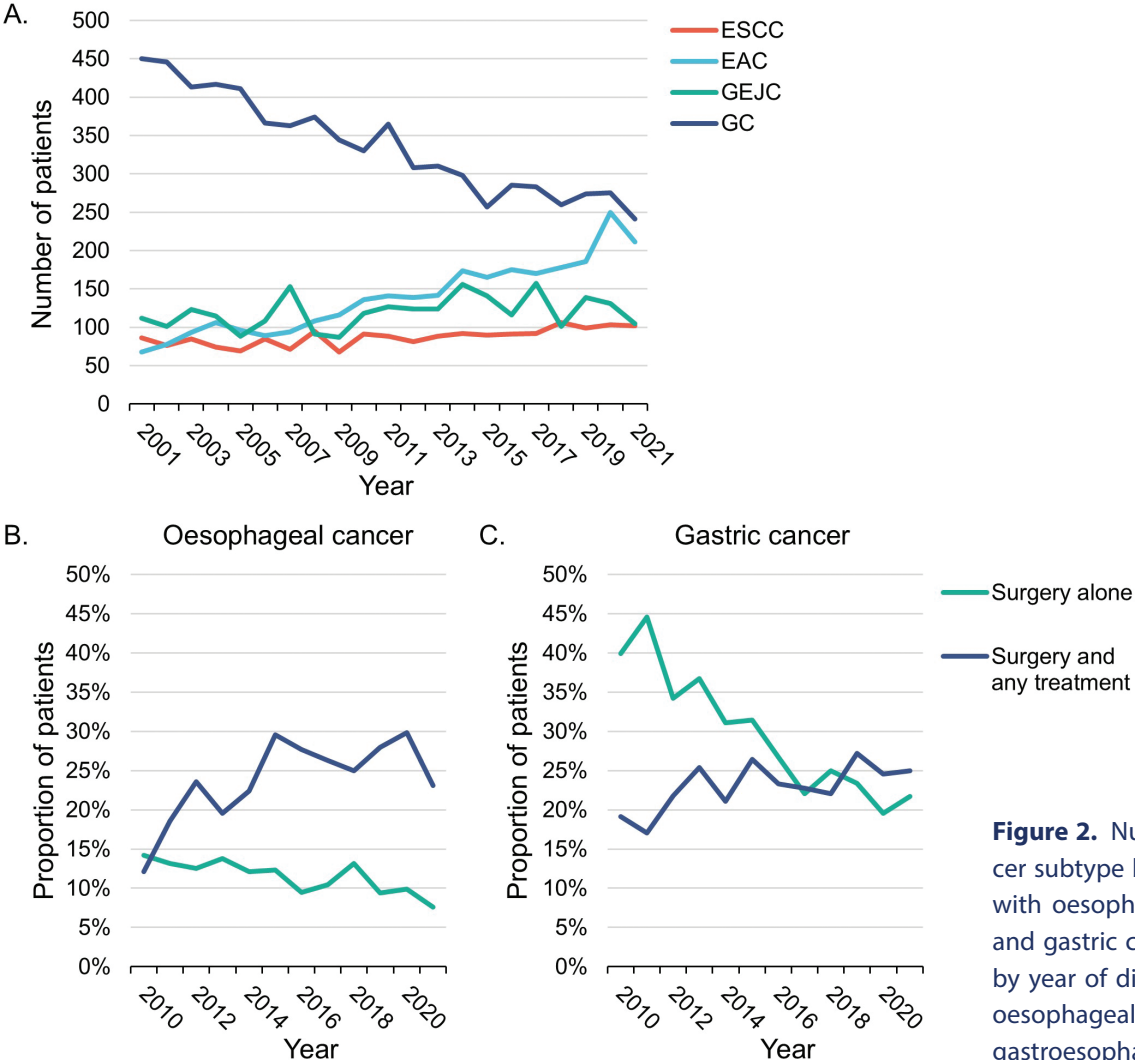
Number of patients with each gastroesophageal cancer subtype by year of diagnosis, (A) and proportion of patients with oesophageal cancer (ESCC, EAC, and GEJC combined) (B) and gastric cancer (C) who received surgery as initial treatment by year of diagnosis. EAC: oesophageal adenocarcinoma; ESCC: oesophageal squamous cell carcinoma; GC: gastric cancer; GEJC: gastroesophageal junction cancer.

Most patients were male, ranging from 56% for GC to 81% for EAC, with a mean age at diagnosis of 69 years for ESCC, EAC, and GEJC, and 73 years for GC (Table 1). Over time the incidence of GC decreased, whereas EAC increased (2001–2021) (Figure 2A). Less than a fifth of patients across all cancer subtypes had localised disease (14–17%) (Table 1).

**Table 1:** Patient demographic and clinical characteristics for each gastroesophageal cancer subtype.

Cancer subtype			ESCC	EAC	GEJC	GC
**Total cohort 2001–2021**						
Patients, *n*			1,832	2,915	2,517	7,070
Age, mean			69	69	69	73
Male, *n* (%)			1,180 (64.4)	2,374 (81.4)	1,915 (76.1)	3,975 (56.2)
Follow-up, mean (min-max)			9.0 (1.0–236.2)	10.5 (0.6–247.8)	11.1 (0.6–238.7)	10.0 (0.6–251.7)
Cancer stage, *n* (%)	Localised		310 (16.9)	477 (16.4)	355 (14.1)	1,178 (16.7)
	Regional		558 (30.5)	839 (28.8)	813 (32.3)	1,999 (28.3)
	Metastatic disease		373 (20.4)	848 (29.1)	814 (32.3)	2,390 (33.8)
	Unknown		591 (32.3)	751 (25.8)	535 (21.3)	1,503 (21.3)
ECOG^‡^, *n* (%)	0		67 (9.8)	185 (13.9)	128 (14.4)	230 (12.3)
	1		252 (36.9)	536 (40.2)	382 (42.9)	637 (34.0)
	2		34 (5.0)	68 (5.1)	50 (5.6)	124 (6.6)
	3		20 (2.9)	36 (2.7)	35 (3.9)	74 (4.0)
	4		1 (0.2)	5 (0.4)	5 (0.6)	13 (0.7)
	Missing		309 (45.2)	505 (37.8)	290 (32.6)	797 (42.5)
**Initial treatment patterns cohort 2010–2021**						
Treatment, *n* (%)	Curative		453 (40.4)	796 (38.6)	595 (38.7)	1,267 (36.4)
	Palliative		433 (38.7)	787 (38.1)	532 (34.6)	867 (24.9)
	No registered treatment		234 (20.9)	482 (23.3)	411 (26.7)	1,347 (38.7)
Survival status for no registered treatment at 12 months, *n* (%)		Alive	26 (11.1)	63 (13.1)	67 (16.3)	187 (13.9)
		Dead	208 (88.9)	419 (86.9)	344 (83.7)	1,160 (86.1)
**Curative treatment**						
Age, mean years			65	65	66	70
Male, *n* (%)			271 (59.8)	693 (87.1)	481 (80.8)	780 (61.6)
Cancer stage, *n* (%)	Localised		85 (18.8)	217 (27.3)	141 (23.7)	331 (26.1)
	Regional		204 (45)	420 (52.8)	374 (62.9)	741 (58.5)
	Metastatic disease		41 (9.1)	63 (7.9)	36 (6.1)	118 (9.3)
	Unknown		123 (27.2)	96 (12.1)	44 (7.4)	77 (6.1)
ECOG^‡^, *n* (%)	0		51 (11.3)	131 (16.5)	90 (15.1)	160 (12.6)
	1		152 (33.6)	326 (41)	235 (39.5)	374 (29.5)
	2		11 (2.4)	20 (2.5)	17 (2.9)	38 (3)
	3		1 (0.2)	3 (0.4)	3 (0.5)	4 (0.3)
	4		0 (0)	0 (0)	0 (0)	0 (0)
	Missing		238 (52.5)	316 (39.7)	250 (42)	691 (54.5)
Treatment, *n* (%)	nCRT		98 (21.6)	238 (29.9)	77 (12.9)	0 (0)
	Definitive CRT		282 (62.3)	141 (17.7)	30 (5)	5 (0.4)
	nCT		12 (2.6)	195 (24.5)	295 (49.6)	501 (39.5)
	Primary surgery		61 (13.5)	222 (27.9)	193 (32.4)	761 (60.1)
**Palliative treatment**						
Age, mean years			71	69	66	66
Male, *n* (%)			294 (67.9)	656 (83.4)	423 (79.5)	501 (57.8)
Cancer stage, *n* (%)	Localised		37 (8.5)	41 (5.2)	26 (4.9)	43 (5.0)
	Regional		118 (27.3)	128 (16.3)	80 (15.0)	98 (11.3)
	Metastatic disease		109 (25.2)	350 (44.5)	267 (50.2)	483 (55.7)
	Unknown		169 (39.0)	268 (34.1)	159 (29.9)	243 (28.0)
ECOG^‡^, *n* (%)	0		16 (3.7)	53 (6.7)	36 (6.8)	61 (7.0)
	1		90 (20.8)	179 (22.7)	129 (24.2)	174 (20.1)
	2		15 (3.5)	20 (2.5)	9 (1.7)	30 (3.5)
	3		8 (1.8)	5 (0.6)	4 (0.8)	2 (0.2)
	4		0	0	0	0
	Missing		304 (70.2)	530 (67.3)	354 (66.5)	600 (69.2)
Treatment, *n* (%)	RT		308 (71.1)	247 (31.4)	82 (8.7)	74 (3.3)
	CRT		61 (14.1)	159 (20.2)	87 (9.2)	96 (4.3)
	CT		64 (14.8)	381 (48.4)	363 (38.5)	697 (31.5)
**Surgery**						
Surgery only, not followed by chemotherapy or radiotherapy			44 (29.5)	194 (31.8)	171 (32.5)	645 (57.0)
Chemotherapy or radiotherapy plus chemotherapy followed by surgery^†^			105 (70.4)	415 (68.1)	355 (67.4)	487 (43.0)

CT: chemotherapy; CRT: chemoradiotherapy; EAC: oesophageal adenocarcinoma; ECOG: Eastern Cooperative Oncology Group; ESCC: oesophageal squamous cell carcinoma; GEJC: gastroesophageal junction cancer; GC: gastric cancer; nCT: neoadjuvant chemotherapy; nCRT: neoadjuvant chemoradiotherapy; RT: radiotherapy.

‡ ECOG is presented from 2015 onwards due to a high proportion of missingness.

† No restriction on same day for chemotherapy or radiotherapy.

#### Initial treatment patterns cohort

The overall proportion of patients who were treated with curative intent was 36–40% across cancer subtypes, with the remainder receiving palliative or no registered treatment (Table 1). A higher proportion of patients with ESCC, EAC, and GEJC were treated palliatively (35–39%) as opposed to those with no registered treatment (21–27%), whereas a lower proportion of patients with GC were treated palliatively (25%), with an increased proportion with no registered treatments (39%) (Table 1). Over 84% of patients with no registered treatment died within 12 months of diagnosis (Table 1).

### Initial treatment patterns

The proportion of patients with oesophageal cancer (ESCC, EAC, and GEJC) who received neoadjuvant chemotherapy or neoadjuvant chemoradiotherapy (surgery and any other treatment) increased over time (2010–2021), whereas the proportion receiving primary surgery (surgery alone) decreased (Figure 2B). The proportion of patients with GC who received primary surgery decreased over time (Figure 2C).

#### Curative treatment

Patients with ESCC treated with curative intent predominantly received definitive chemoradiotherapy (62%) and neoadjuvant chemoradiotherapy (22%) (Table 1). For GEJC, 50% of patients received neoadjuvant chemotherapy compared with 25% of patients with EAC, while the proportion of patients who received primary surgery was comparable between the two subtypes (32 and 28%, respectively) (Table 1). Most patients with GC received primary surgery (60%) or neoadjuvant chemotherapy (40%) (Table 1).

Most patients aged 70 years of age or over who were treated with curative intent for GC (78%), GEJC (52%), and EAC (43%) underwent primary surgery (Supplementary Table S4). Patients under 70 years predominantly received neoadjuvant chemotherapy for GC (63%) and GEJC (59%), or neoadjuvant chemoradiotherapy for EAC (35%) (Supplementary Table S4). Definitive chemoradiotherapy was the most common treatment for ESCC, regardless of age (< 70 years, 61% and ≥ 70 years, 65%) (Supplementary Table S4).

Regional disease was equally distributed between patients with EAC and GEJC who received neoadjuvant chemotherapy and neoadjuvant chemoradiotherapy (67–74%) (Supplementary Table S5). Patients with EAC, GEJC, and GC receiving primary surgery had localised disease in 55, 41, and 30% of cases, respectively (Supplementary Table S5).

#### Palliative treatment

Patients with ESCC treated palliatively primarily received radiotherapy (71%) (Table 1). A third to a half of patients with EAC (48%), GEJC (39%), and GC (32%) received chemotherapy, versus 15% of patients with ESCC (Table 1).

### Overall survival

#### Total cohort

**Figure 3:**
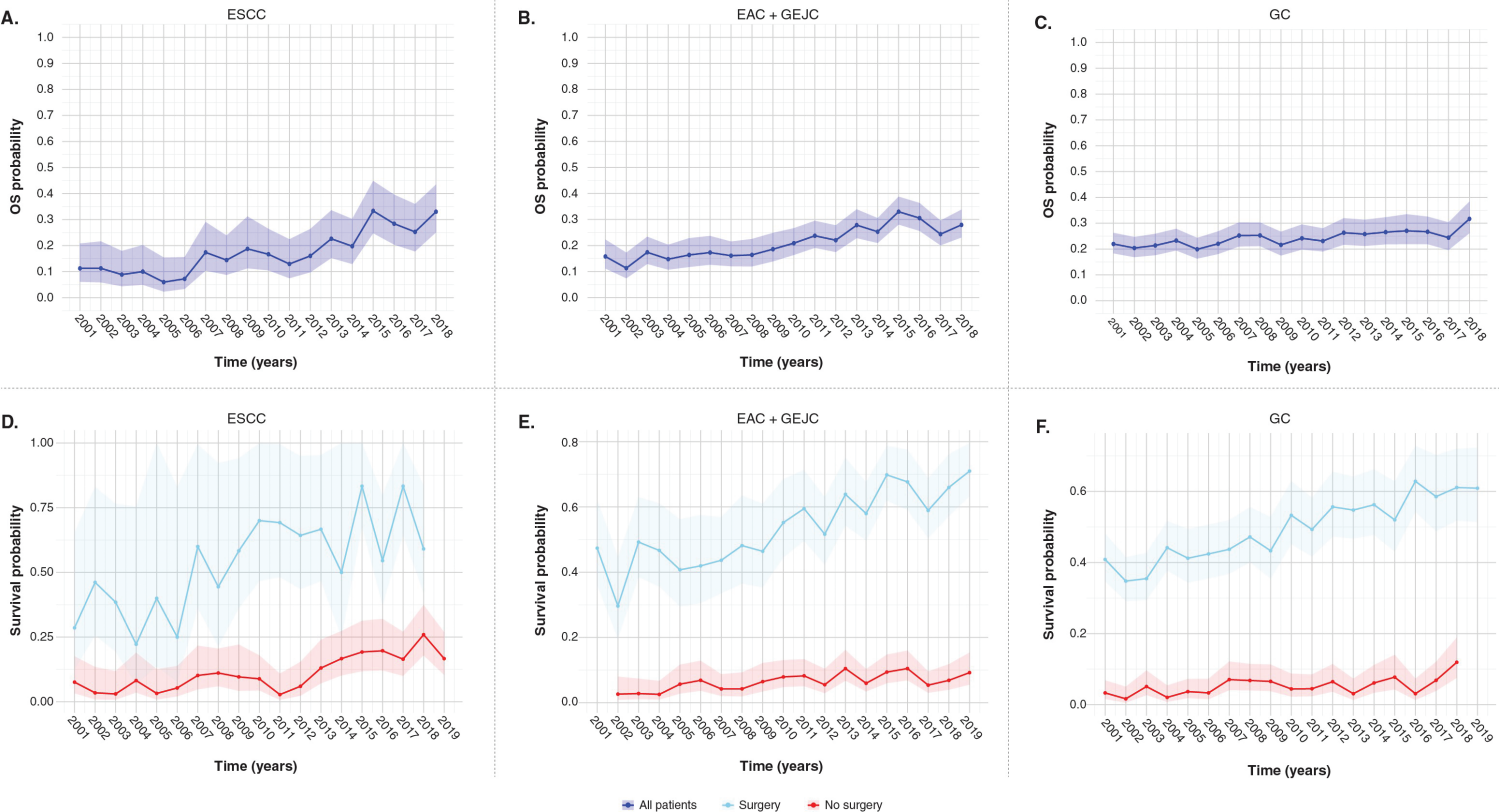
Three-year survival (95% CI) for patients with (A, D) ESCC, (B, E) combined EAC and GEJC, and (C, F) GC, for all patients and stratified by whether they received surgery. CI: confidence interval; EAC: oesophageal adenocarcinoma; ESCC: oesophageal squamous cell carcinoma; GEJC: gastroesophageal junction cancer; GC: gastric cancer; OS: overall survival.

Three-year survival was observed to improve for patients in all cancer subtypes from 2001 to 2018 (Figure 3A–C). When stratified by whether a patient received surgery or not, the improvement in 3-year survival was predominantly in those patients who received surgery (Figure 3D–E).

Kaplan–Meier survival analysis from 2001 to 2021 demonstrated median OS ranging from 9.0 months [95% CI: 8.0, 10.0] (ESCC) to 12.0 months [95% CI: 11.1, 12.1] (EAC) across all subtypes (Supplementary Figure S1; Table 2). Survival decreased in the < 70-year population across all cancer subtypes (Supplementary Table S6; Supplementary Figure S2).

**Table 2:** Median OS and Cox regression multivariate analysis for patients with each gastroesophageal cancer subtype and those stratified by initial treatment strategy.

		Median OS									
		ESCC		EAC		GEJC		GC		EAC + GEJC	
**Total cohort 2001–2021**		**Months**	**95% CI**	**Months**	**95% CI**	**Months**	**95% CI**	**Months**	**95% CI**	**Months**	**95% CI**
		9.0	8.1, 10.0	12.0	11.1, 12.1	12.0	11.0, 13.0	10.1	10.0, 11.0	NA	NA
**Initial treatment patterns cohort 2010–2021**		**Months**	**95% CI**	**Months**	**95% CI**	**Months**	**95% CI**	**Months**	**95% CI**	**Months**	**95% CI**
Curative treatment	nCRT	87.1	59.1, NE	49.1	39.0, 75.1	42.1	35.1, NE	-	-	NA	NA
	Definitive CRT	24.0	18.1, 32.0	17.0	14.1, 21.1	17.6	13.0, 30.1	41.1	15.1, NE	NA	NA
	nCT	94.4	20.1, NE	86.1	51.1, NE	75.1	49.1, NE	63.1	49.1, 105.1	NA	NA
	Primary surgery	45.0	31.0, NE	95.1	67.1, NE	41.0	33.1, 56.1	39.1	35.0, 49.0	NA	NA
Palliative treatment	RT	7.1	6.0, 8.1	8.0	7.0, 9.1	6.1	6.0, 10.1	8.0	5.0, 9.1	NA	NA
	CRT	8.1	7.1, 11.0	10.0	9.1, 11.0	10.1	9.0, 13.0	9.0	8.0, 10.0	NA	NA
	CT	8.0	5.1, 10.1	9.1	8.1, 11.0	11.0	10.0, 12.0	10.0	9.0, 11.0	NA	NA
No registered treatment^‡^		3.1	2.1, 4.0	3.1	3.0, 4.1	3.1	3.0, 4.0	3.1	3.1, 4.0	NA	NA
**Cox regression**											
**Initial treatment patterns cohort 2010–2021**		**HR**	**95% CI**	**HR**	**95% CI**	**HR**	**95% CI**	**HR**	**95% CI**	**HR**	**95% CI**
Female (reference male)		0.72	0.55, 0.94	1.09	0.80, 1.48	0.82	0.60, 1.11	0.98	0.84, 1.14	0.96	0.77, 1.19
Age, mean years		1.01	1.00, 1.03	1.02	1.01, 1.03	1.02	1.01, 1.03	1.03	1.02, 1.04	1.02	1.01, 1.03
Cancer stage (reference localised)	Metastatic disease	2.06	1.24, 3.43	4.10	2.68, 6.29	5.62	3.42, 9.26	7.58	5.73, 10.03	4.73	3.43, 6.53
	Regional	1.44	0.97, 2.13	2.58	1.90, 3.51	2.65	1.92, 3.66	2.97	2.40, 3.66	2.69	2.16, 3.36
	Unknown	1.32	0.84, 2.05	1.58	1.05, 2.37	1.54	0.92, 2.60	1.65	1.10, 2.46	1.58	1.15, 2.17
Curative treatment (reference nCT)	nCRT	1.03	0.43, 2.46	1.54	1.11, 2.13	1.45	1.02, 2.08	0	0	1.42	1.15, 1.76
	Definitive CRT	2.07	0.89, 4.81	3.45	2.44, 4.90	3.27	2.06, 5.18	2.61	0.96, 7.08	3.22	2.51, 4.12
	Primary surgery	1.45	0.60, 3.49	1.33	0.92, 1.92	1.73	1.31, 2.28	1.22	1.02, 1.46	1.50	1.20, 1.86

Note: Reference variables were sex (male), cancer stage (localised), and curative treatment (neoadjuvant chemotherapy).

CI: confidence interval; CT: chemotherapy; CRT: chemoradiotherapy; EAC: oesophageal adenocarcinoma; ESCC: oesophageal squamous cell carcinoma; GEJC: gastroesophageal junction cancer; GC: gastric cancer; HR: hazard ratio; nCT: neoadjuvant chemotherapy; nCRT: neoadjuvant chemoradiotherapy; NE: not estimable; OS: overall survival; RT: radiotherapy.

‡ No treatment registered in the 12 months following diagnosis.

#### Initial treatment patterns cohort

**Figure 4:**
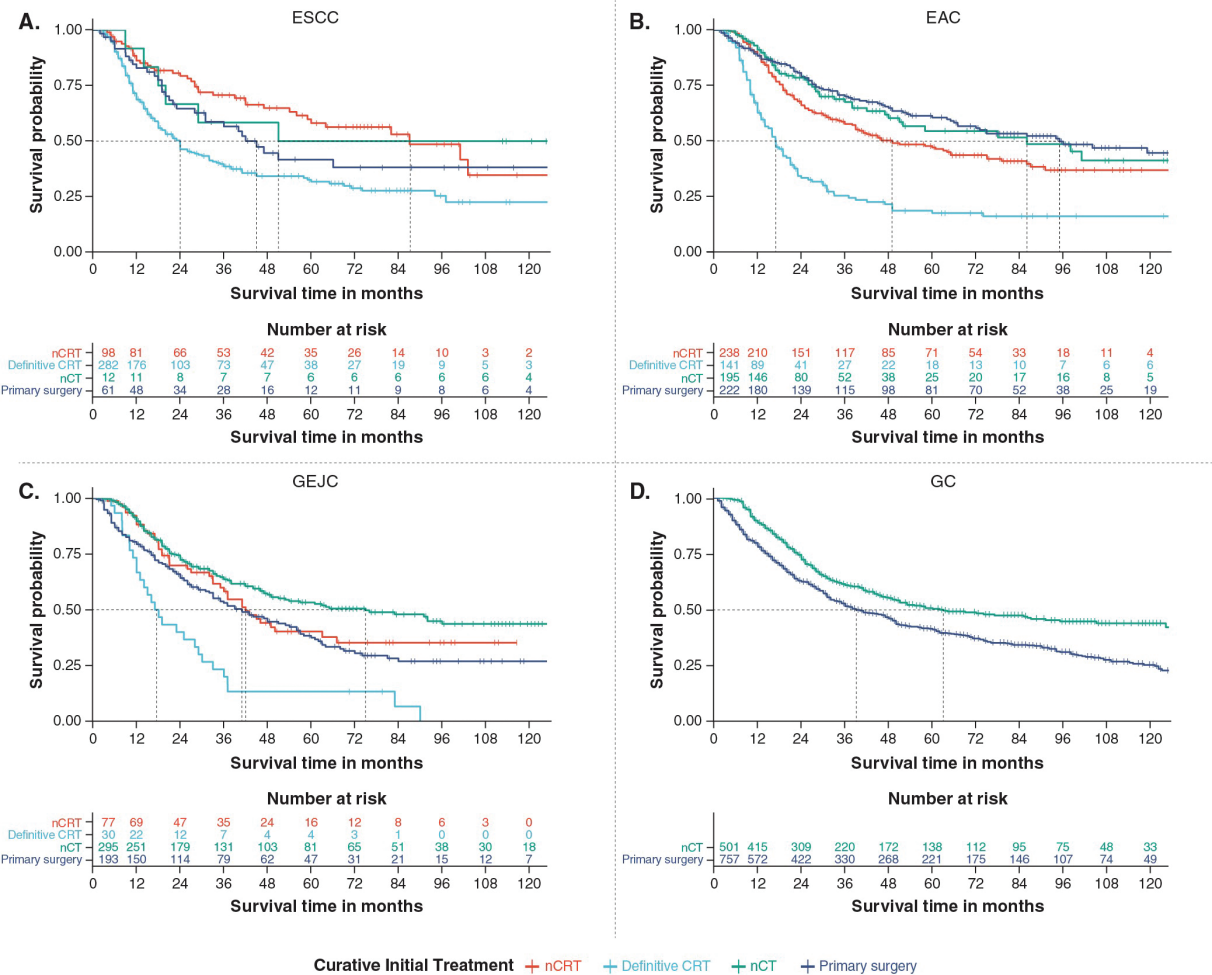
Median OS for patients with (A) ESCC, (B) EAC, (C) GEJC, and (D) GC stratified by initial curative treatment strategy. CRT: chemoradiotherapy; EAC: oesophageal adenocarcinoma; ESCC: oesophageal squamous cell carcinoma; GEJC: gastroesophageal junction cancer; GC: gastric cancer; nCT: neoadjuvant chemotherapy; nCRT: neoadjuvant chemoradiotherapy; OS: overall survival.

Median OS from 2010 to 2021 in patients treated with curative intent varied by cancer subtype and treatment strategy, ranging from 17.0 months [95% CI: 14.1, 21.1] in patients with EAC who received definitive chemoradiotherapy, to 95.1 months [95% CI: 67.1, not estimable (NE)] in patients with EAC who received primary surgery (Figure 4; Table 2). Patients with GEJC and GC receiving primary surgery had a median OS of 41.0 months [95% CI: 33.1, 56.1] and 39.1 months [95% CI: 35.0, 49.0]. Patients with EAC and GEJC treated with neoadjuvant chemoradiotherapy had a median OS of 49.1 months [95% CI: 39.0, 75.1] and 42.1 months [95% CI: 35.1, NE], respectively, versus 86.1 months [95% CI: 51.1, NE] and 75.1 months [95% CI: 49.1, NE] in those treated with neoadjuvant chemotherapy (Figure 4; Table 2). Patients with GC receiving neoadjuvant chemotherapy had a median OS of 63.1 months [95% CI: 49.1, 105.1].

Median OS from 2010 to 2021 for patients treated palliatively ranged from 6.1 [95% CI:6.0, 10.1] months in patients with GEJC receiving radiotherapy, to 11.0 [95% CI:10.0, 12.0] months in patients with GEJC receiving chemotherapy (Table 2).

**Figure 5:**
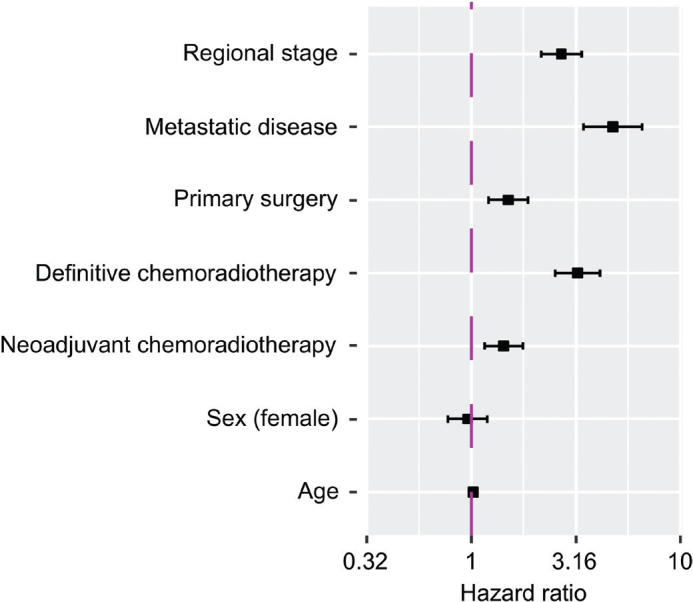
Forest plot depicting the Cox regression multivariate analysis of overall survival in the combined patients with EAC or GEJC treated with curative intent. Note: The Forest plot illustrates the hazard ratio for mortality within the time period of the initial treatment patterns cohort 2010–2021. Reference variables were sex (male), cancer stage (localised), and curative treatment (neoadjuvant chemotherapy). EAC: oesophageal adenocarcinoma, GEJC: gastroesophageal junction cancer.

A multivariate Cox regression model, adjusted for age, sex, and disease stage, demonstrated lower survival with neoadjuvant chemoradiotherapy compared with neoadjuvant chemotherapy in patients with EAC, GEJC, and combined EAC or GEJC (hazard ratio (HR): 1.5 [95% CI: 1.1, 2.1], 1.5 [95% CI: 1.0, 2.1], and 1.4 [95% CI: 1.2, 1.8], respectively) (Table 2; Figure 5).

## Discussion

This study describes the demographics and long-term trends in treatment patterns and survival outcomes for Norwegian patients with ESCC, EAC, GEJC, and GC, based on a large population-level database. The demographics of the Norwegian population broadly align to global estimates, with an overall increase in gastroesophageal cancer incidence that disproportionately affects men [21–23]. A notable proportion of patients had metastatic disease, with less than a fifth of patients diagnosed with localised disease. This reflects the typical advanced disease diagnosis, with intra- and inter-tumoral heterogeneity [24]. and represents a barrier to curative treatment.

Approximately 40% of Norwegian patients were treated with curative intent, with treatment patterns broadly aligned to the current ESMO clinical practice guidelines for gastric and oesophageal cancer [13, 14]. A shift in treatment patterns was observed from 2010 to 2021, with a move from primary surgery to multi-modal treatment using neoadjuvant chemotherapy and neoadjuvant chemoradiotherapy. This aligns with a shift in treatment patterns over time reported previously, and highlights the changing role of surgery [25]. Additionally, in the latter part of the study period, an increase in the treatment of oligometastatic disease was observed. The increase in multi-modality treatments likely reflects changes to the treatment landscape following the pivotal MAGIC, CROSS, and FLOT clinical trials [10, 12]. Despite this increase, we observe that patients with GC have the highest proportion primary surgery as initial treatment, most likely due to advanced age and comorbidities. In our study, a notable increase in 3-year survival was seen from 2008, which corresponds with the adoption of multimodal treatment in Norway following these trials [10, 12].

This study reports median OS of 9.0–12.0 months for patients with gastroesophageal cancer in Norway, decreasing with metastatic disease and aligning with published estimates from randomised control trials [26]. In patients treated with curative intent, survival varied by cancer subtype and treatment strategy, with median OS ranging from 17.0 to 95.1 months in patients with EAC who received definitive chemoradiotherapy compared with primary surgery.

In patients with EAC and GEJC, those treated with neoadjuvant chemotherapy demonstrated a median OS nearly twice that of those receiving neoadjuvant chemoradiotherapy. This finding supports the results of recent studies. In the ESOPEC trial, improved outcomes were demonstrated with the perioperative FLOT regimen versus the tri-modality CROSS regimen, although the CROSS arm underperformed in this trial [15]. A recent retrospective study in a US population also found similar results, with increased survival among patients receiving perioperative chemotherapy compared to neoadjuvant chemoradiotherapy [27]. However, the study was limited by a small population for the perioperative chemotherapy group, and it included both patients with ESCC and EAC in the analysis. Additionally, the study did not conduct an adjusted analysis to directly compare perioperative chemotherapy and neoadjuvant chemoradiotherapy. Interestingly, the neo-AEGIS trial demonstrated equivalent survival outcomes between perioperative chemotherapy and perioperative chemoradiotherapy; however, this study was underpowered and terminated early, and the majority of patients in the perioperative chemotherapy arm received the MAGIC regimen which was already known to be inferior to FLOT [28]. Together, these results question the role of radiotherapy in the neoadjuvant curative setting of gastroesophageal adenocarcinoma. Nonetheless, caution must be exercised due to the retrospective method of our study and the potential risk for bias and confounding.

Patients with EAC who underwent primary surgery demonstrated the highest median OS. In the adjusted models, neoadjuvant chemotherapy was not significantly superior to primary surgery for EAC, compared to GEJC and GC. This may be due to the broad definition of localised disease used in our study, which prevented us from adjusting for the most localised cancers amenable for endoscopic surgery. Lower median OS was observed among patients with GEJC and GC treated with primary surgery, but these groups contained more locoregional disease and older patients, who were possibly deemed not fit to receive chemotherapy. Patients with ESCC receiving neoadjuvant chemoradiotherapy had higher median OS compared to EAC and GEJC patients, although stage and age were equally distributed. The superior survival among ESCC patients aligns with the updated long-term survival data from the CROSS trial [29].

This study has several strengths. Firstly, it is based on a national population-level database, with a large cohort representative of the Nordic population [1, 2]. Secondly, the 10-year period of treatment patterns follows patients over their complete follow-up time to discern long-term trends and outcomes. Lastly, the results are stratified by four subtypes of gastroesophageal cancer, and curative or palliative treatments, to provide a comprehensive view of real-world gastroesophageal cancer management in Norway.

This study has limitations inherent to RWE population-based analysis. Firstly, it is a descriptive overview of long-term trends in gastroesophageal cancer. Due to the broad treatment landscape and the advent of new therapies (e.g. immunotherapy) and diagnostic tests, this study does not stratify patients by different types/regimens of chemotherapy, immunotherapy, or diagnostic tests. Secondly, the study is retrospective and does not encapsulate clinical and patient factors, with treatment likely based on disease staging, or patient fitness, which may be independent factors for the likelihood of survival. Therefore, it is likely that selection bias may have influenced the results, with more frail patients being selected for neoadjuvant chemoradiotherapy, definitive chemoradiotherapy, and primary surgery instead of receiving chemotherapy. We also had varying degrees of missing data. No data was available on clinical or pathological TNM (tumour, node, metastasis) staging, only U.S. National Cancer Institute’s SEER program, which can be imprecise, as stages can be relatively heterogeneous. Lastly, the potential survival impact of the recent introduction of adjuvant immunotherapy with nivolumab after neoadjuvant chemoradiotherapy for oesophageal cancer is not reflected in the current RWE study [30].

## Conclusion

This study provides a valuable insight into the long-term trends in management and patient outcomes for gastroesophageal cancer in Norway. Importantly, patients with EAC and GEJC who received neoadjuvant chemotherapy demonstrated improved survival compared with those who received neoadjuvant chemoradiotherapy. Future studies on the impact of newer innovative therapies on survival are required to identify optimum treatment strategies for each gastroesophageal cancer subtype.

## Supplementary Material

Long-term trends in the clinical management and outcomes of patients with gastroesophageal cancer in Norway

## Data Availability

The study has used data from the Cancer Registry of Norway. The data cannot be shared according to regulations given by the Regional Ethics Committee (reference: 339296) and the Cancer Registry of Norway (reference DU-3862 22/70).
