# Supplementary material for: Long-term trends in the clinical management and outcomes of patients with gastroesophageal cancer in Norway
Source: Acta Oncol. 2025 Apr 15;64:43167. doi: 10.2340/1651-226X.2025.43167 (PMC12016665; doi:10.2340/1651-226X.2025.43167)
Supplement: Long-term trends in the clinical management and outcomes of patients with gastroesophageal cancer in Norway [file AO-64-43167-s1.pdf]

Supplementary material has been published as submitted. It has not been copyedited, or typeset by Acta Oncologica

SUPPLEMENTARY TABLES AND FIGURES

Table S1. Topography and morphology codes defining cancer subtype populations

Patient group	Topography	Morphology
ESCC	C15.0–C15.9 and C16.0	8032, 8033, 8050-8084, 8575, 8980
EAC	C15.0–C15.9	8140-8145, 8190, 8201, 8210, 8211, 8230,8255, 8260-8263, 8310,8323, 8401, 8471, 8480, 8481, 8490, 8510, 8512, 8530, 8560, 8570-8574, 8576
GEJC	C16.0	8140-8145, 8190, 8201, 8210, 8211, 8230,8255, 8260-8263, 8310,8323, 8401, 8471, 8480, 8481, 8490, 8510, 8512, 8530, 8560, 8570-8573, 8576
GC	C16.1–C16.9	8140-8145, 8190, 8201, 8210, 8211, 8230,8255, 8260-8263, 8310,8323, 8401, 8471, 8480, 8481, 8490, 8510, 8512, 8530, 8560, 8570-8573, 8576

Abbreviations: EAC, oesophageal adenocarcinoma; ESCC, oesophageal squamous cell carcinoma; GEJC, gastroesophageal junction cancer; GC, gastric cancer

Table S2. Treatment algorithm

Group	Sequence of initial treatments
Initial curative treatment	Neoadjuvant chemoradiotherapy – chemotherapy plus radiotherapy on same date (any dose) followed by surgery within 12 months of diagnosis
	Definitive chemoradiotherapy – chemotherapy plus radiotherapy (≥ 40 Gy) (whether on the same day or not) and no surgery within 12 months diagnosis
	Neoadjuvant chemotherapy – chemotherapy followed by surgery within 12 months of diagnosis, no radiotherapy before surgery but can occur on the same day or following surgery
	Primary surgery
Initial palliative treatment	Radiotherapy (any dose and no chemotherapy or surgery within 12 months of diagnosis)
	Chemoradiotherapy (chemotherapy plus radiotherapy (< 40 Gy) and no surgery within 12 months)
	Chemotherapy and no surgery or radiotherapy within 12 months of diagnosis
No registered treatment	No surgery, chemotherapy, or radiotherapy
Other	Patient whose first treatment post diagnosis did not fall into the above categories
Surgery-focus treatment groups	Primary surgery only
	Chemotherapy or chemotherapy plus radiotherapy followed by surgery[†]

†No restriction on same day for chemotherapy or radiotherapy
Abbreviations: Gy, Gray

Table S3. Study variables and endpoints

Variables	Definition	Timings
**Demographic characteristics**		
Age	Age at index date	At index date
Sex	• Male<br>• Female	At index date
**Clinical characteristics**		
ECOG scores	Scored from 0 to 4	At index date
**Disease history**		
Cancer type	• Oesophageal cancer<br>• Gastric cancer	At index date
Cancer stage	Categorical variables:<br>• Local<br>• Regional<br>• Metastatic disease<br>• Unknown	At index date
**Metastasis at diagnosis**	• Yes – if so, site<br>• No	At index date
**Treatment patterns**		
**Surgery**	• No<br>• Yes, if yes: date of surgery	Follow up period
Radiotherapy	• No<br>• Yes, if yes: dates, and type < 40 Gy (palliative)/> 40 Gy (curative)	Follow up period
Chemotherapy	• No<br>• Yes, if yes: dates	Follow up period
**Disease outcomes**		
Overall survival	Time from start of primary diagnosis (index date) until date of death or date patient was last known to be alive (if lost to follow-up)	Follow-up

Abbreviations: ECOG, Eastern Cooperative Oncology Group

Table S4. Initial treatment patterns for patients with each gastroesophageal cancer subtype stratified by age

Initial treatment patterns cohort 2010–2021		Age, years old							
		< 70	≥ 70	< 70	≥ 70	< 70	≥ 70	< 70	≥ 70
		ESCC		EAC		GEJC		GC	
Treatment, n (%)	Curative	295 (53.3)	158 (28.0)	512 (49.8)	284 (27.5)	348 (45.7)	247 (31.9)	530 (42.6)	737 (33.0)
	Palliative	188 (33.9)	245 (43.3)	407 (39.5)	380 (36.7)	320 (42.0)	212 (27.3)	497 (39.9)	370 (16.6)
	No registered treatment	71 (12.8)	163 (28.8)	111 (10.8)	371 (35.8)	94 (12.3)	317 (40.9)	219 (17.6)	1,128 (50.5)
**Curative treatment**		ESCC		EAC		GEJC		GC	
Treatment, n (%)	nCRT	74 (25.1)	24 (15.2)	180 (35.2)	58 (20.5)	58 (16.7)	19 (7.7)	0	0
	Definitive CRT	179 (60.7)	103 (65.2)	96 (18.8)	45 (15.9)	21 (6.1)	9 (3.7)	4 (0.8)	1 (0.2)
	nCT	9 (3.1)	3 (1.9)	136 (26.6)	59 (20.8)	205 (59.0)	90 (36.5)	336 (63.4)	165 (22.4)
	Primary surgery	33 (11.2)	28 (17.8)	100 (19.6)	122 (43.0)	64 (18.4)	129 (52.3)	190 (35.9)	571 (77.5)
**Palliative treatment**		ESCC		EAC		GEJC		GC	
Treatment, n (%)	RT	98 (52.2)	210 (85.8)	58 (14.3)	189 (49.8)	17 (5.4)	65 (30.7)	13 (2.7)	61 (16.5)
	CRT	43 (22.9)	18 (7.4)	110 (27.1)	49 (12.9)	58 (18.2)	29 (13.7)	67 (13.5)	29 (7.9)
	CT	47 (25.0)	17 (7.0)	239 (58.8)	142 (37.4)	245 (76.6)	118 (55.7)	417 (84.0)	280 (75.7)

Abbreviations: CT: chemotherapy; CRT, chemoradiotherapy; EAC: oesophageal adenocarcinoma; ESCC: oesophageal squamous cell carcinoma; GC: gastric cancer; GEJC: gastroesophageal junction cancer; nCRT: neoadjuvant chemoradiotherapy; nCT: neoadjuvant chemotherapy; RT: radiotherapy

Table S5. Initial treatment patterns for patients with each gastroesophageal cancer subtype stratified by cancer stage

Cancer stage	nCRT	Definitive CRT	nCT	Primary surgery
**ESCC**				
Localised	25 (25.5)	33 (11.7)	4 (33.3)	23 (37.7)
Regional	63 (64.3)	98 (34.8)	8 (66.7)	35 (57.4)
Metastatic disease	4 (4.1)	36 (12.8)	0 (0)	1 (1.6)
Unknown	6 (6.1)	115 (40.8)	0 (0)	2 (3.3)
**EAC**				
Localised	53 (22.3)	15 (10.6)	27 (13.9)	122 (55.0)
Regional	160 (67.2)	40 (28.4)	144 (73.9)	76 (34.2)
Metastatic disease	12 (5.0)	34 (24.1)	13 (6.7)	4 (1.8)
Unknown	13 (5.5)	52 (36.9)	11 (5.6)	20 (9.0)
**GEJC**				
Localised	16 (20.8)	4 (13.3)	42 (14.2)	79 (40.9)
Regional	54 (70.1)	9 (30.0)	214 (72.5)	97 (50.3)
Metastatic disease	1 (1.3)	8 (26.7)	19 (6.4)	8 (4.2)
Unknown	6 (7.8)	9 (30)	20 (6.8)	9 (4.7)
**GC**				
Localised	0 (0)	1 (20.0)	103 (20.6)	227 (29.8)
Regional	0 (0)	0 (0)	301 (60.1)	440 (57.8)
Metastatic disease	0 (0)	2 (40.0)	43 (8.6)	73 (9.6)
Unknown	0 (0)	2 (40.0)	54 (10.8)	21 (2.8)

Abbreviations: CRT, chemoradiotherapy; EAC: oesophageal adenocarcinoma; ESCC: oesophageal squamous cell carcinoma; GC: gastric cancer; GEJC: gastroesophageal junction cancer; nCRT: neoadjuvant chemoradiotherapy; nCT: neoadjuvant chemotherapy

Table S6. Median OS for patients with each gastroesophageal cancer subtype stratified by age and cancer stage

Total cohort 2001–2021	Median OS							
	Months	95% CI	Months	95% CI	Months	95% CI	Months	95% CI
	ESCC		EAC		GEJC		GC	
< 70 years	11.01	10.03, 12.03	15.98	14.04, 17.06	16.08	15.06, 17.98	15.06	14.04, 16.08
≥ 70 years	7.07	7.00, 8.05	9.01	8.05, 10.03	8.05	7.07, 9.04	8.05	8.02, 9.01
Localised	12.03	10.0, 17.03	38.04	28.11, 52.01	35.05	27.06, 47.05	47.08	40.08, 61.05
Regional	11.11	10.0, 13.05	19.1	17.06, 21.07	20.09	18.08, 24.03	19.99	19.00, 21.07
Metastatic disease	5.06	4.08, 6.02	6.05	5.98, 7.00	5.98	5.06, 6.08	5.00	4.04, 5.03
Unknown	9.01	8.02, 9.99	9.04	8.05, 10.03	9.01	8.02, 10.06	8.09	7.99, 9.04

Abbreviations: CI, confidence interval; EAC, oesophageal adenocarcinoma; ESCC, oesophageal squamous cell carcinoma; GEJC, gastroesophageal junction cancer; GC, gastric cancer; OS, overall survival

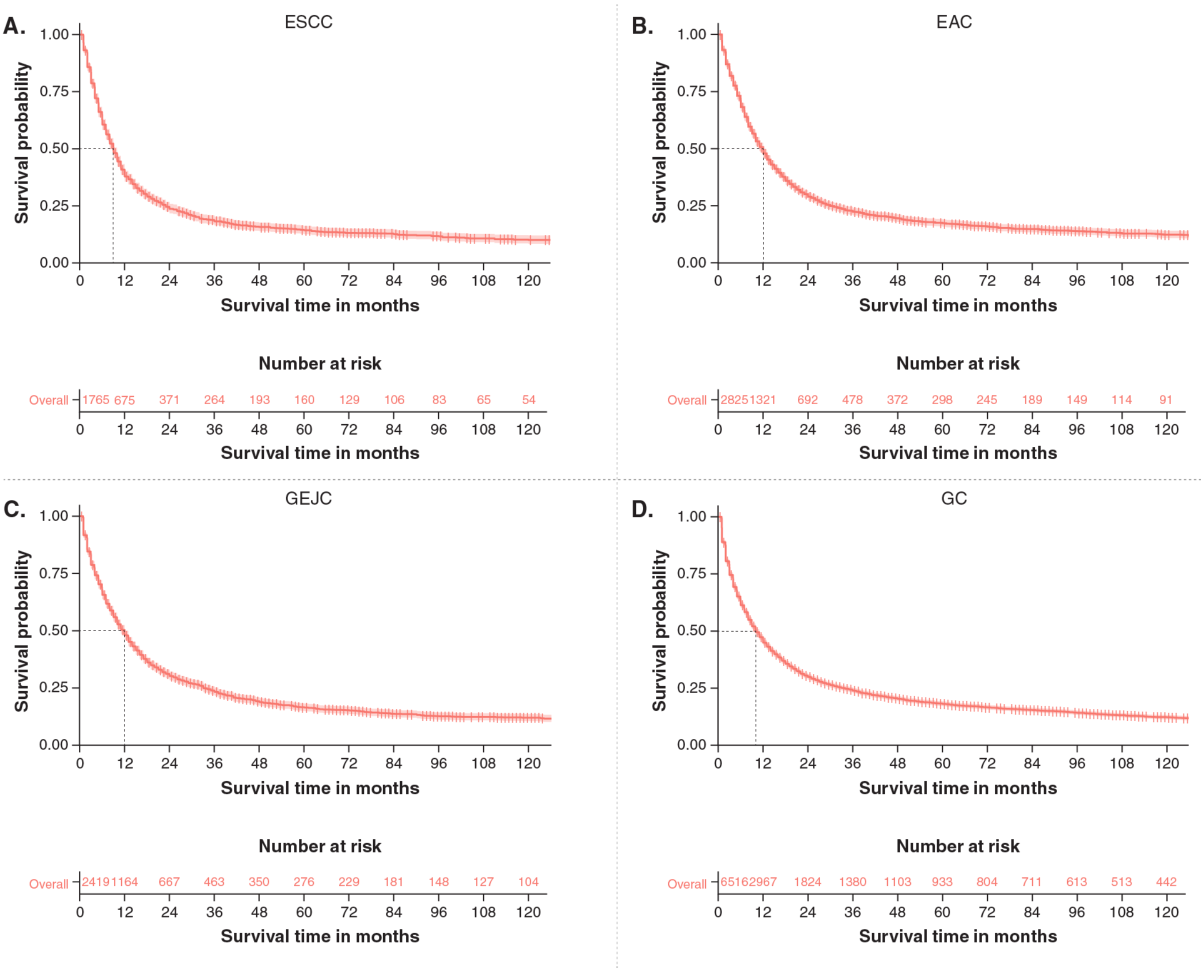

Supplementary Figure S1. Median OS for patients with A) ESCC, B) EAC, C) GEJC, and D) GC

Abbreviations: EAC, oesophageal adenocarcinoma; ESCC, oesophageal squamous cell carcinoma; GEJC, gastroesophageal junction cancer; GC, gastric cancer

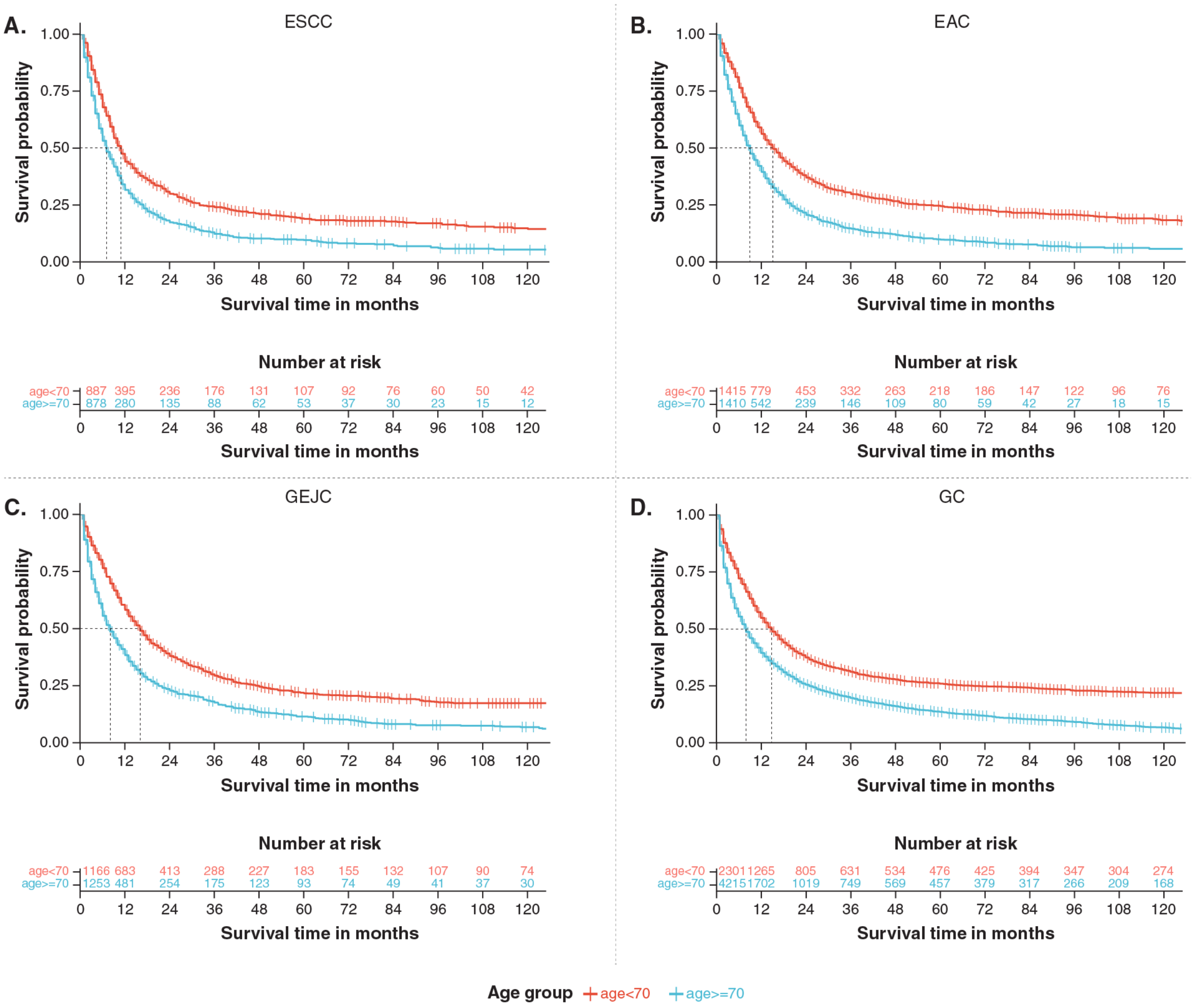

Figure S2. Median OS for patients with A) ESCC, B) EAC, C) GEJC, and D) GC stratified by age

Abbreviations: EAC, oesophageal adenocarcinoma; ESCC, oesophageal squamous cell carcinoma; GEJC, gastroesophageal junction cancer; GC, gastric cancer; OS, overall survival

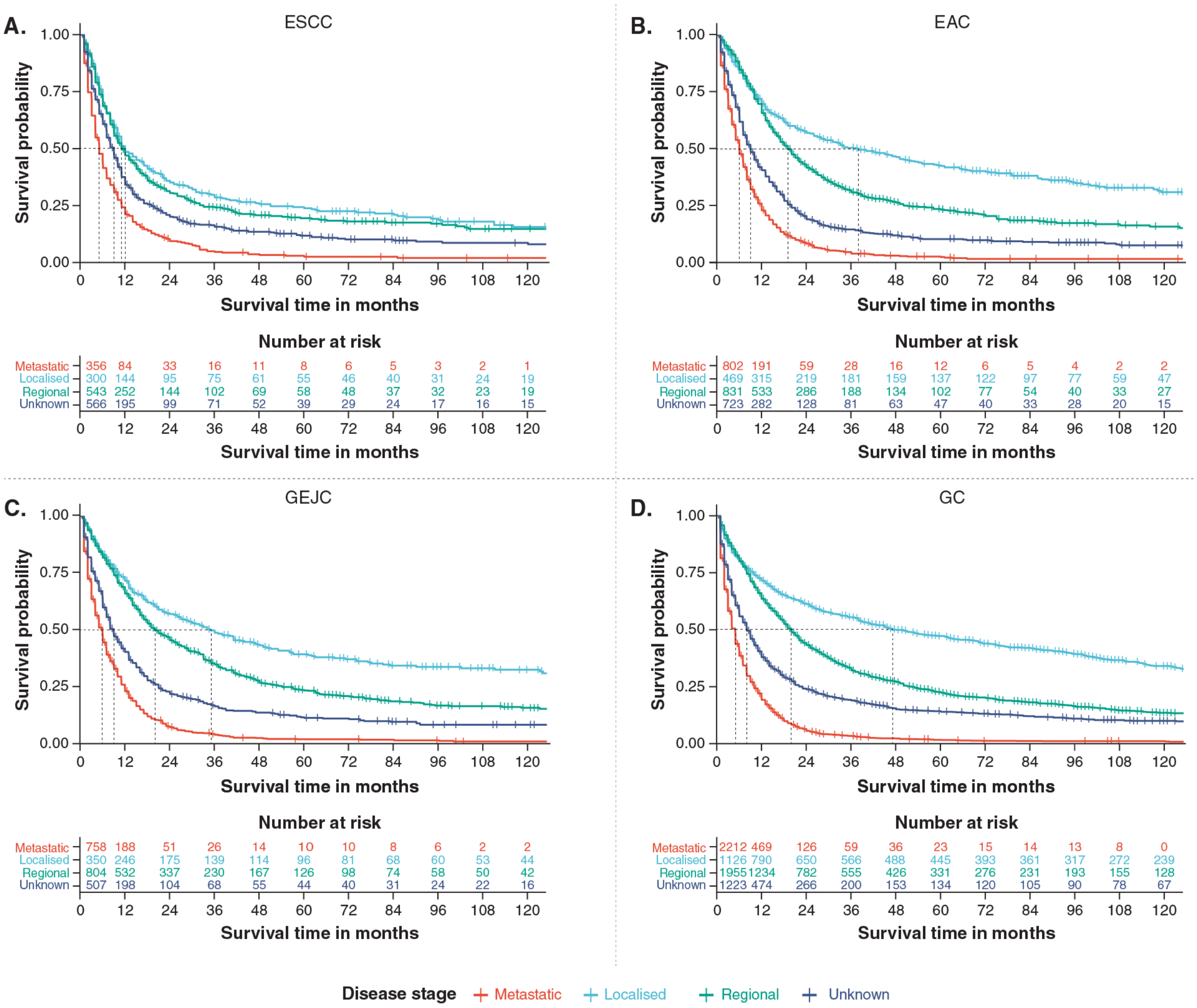

Figure S3. Median OS for patients with A) ESCC, B) EAC, C) GEJC, and D) GC stratified by cancer stage

Abbreviations: EAC, oesophageal adenocarcinoma; ESCC, oesophageal squamous cell carcinoma; GEJC, gastroesophageal junction cancer; GC, gastric cancer; OS, overall survival
